# Positive fertility outcomes in a female with classic congenital adrenal hyperplasia following bilateral adrenalectomy

**DOI:** 10.1186/s13633-016-0028-4

**Published:** 2016-05-20

**Authors:** Urania Dagalakis, Ashwini Mallappa, Meredith Elman, Martha Quezado, Deborah P. Merke

**Affiliations:** National Institutes of Health, Clinical Center, 10 Center Drive Building 10, Rm 1-2740, Bethesda, 20892-1932 MD USA; National Cancer Institute, Bethesda, MD USA; Eunice Kennedy Shriver National Institute of Child Health and Human Development, National Institutes of Health, Bethesda, MD USA; Albany Medical College, Albany, NY USA

**Keywords:** Classic CAH, Fertility outcomes, Bilateral adrenalectomy, Adrenal hyperplasia, Hyperandrogenism

## Abstract

**Background:**

Classic congenital adrenal hyperplasia (CAH) requires lifetime steroid replacement and supraphysiologic glucocorticoid dose is often required for adequate adrenal androgen suppression. Patients often suffer from long-term co-morbidities and female infertility is common.

**Case presentation:**

We report the use of laparoscopic bilateral adrenalectomy as a treatment for a 21 year old female with classic simple virilizing CAH and infertility. She presented as an adolescent with increasing weight gain, amenorrhea and elevated adrenal androgens despite the use of dexamethasone (250 mcg given twice daily), and fludrocortisone (150 mcg daily). An anti-androgen (flutamide 250 mg given twice daily) and a combined oral contraceptive pill were added to her regimen and prevented progressive virilization, but she eventually desired fertility. A bilateral laparoscopic adrenalectomy was performed at age 21. The right adrenal gland weighed 41.8 grams and the left gland 45.5 grams. There were no complications during the surgery.

Since the surgery, she has had a total of three pregnancies, resulting in 3 healthy full-term infants. Follow-up 7 years later at age 27 revealed overall excellent health with a BMI of 25.1 kg/m^2^, no evidence of adrenal rest tissue based on hormonal testing, above average quality-of-life based on 36-item short-form health survey and she has not experienced an adrenal crisis.

**Conclusions:**

This case highlights the use of bilateral adrenalectomy as a treatment option for female infertility in a patient with classic CAH and difficult-to-control hyperandrogenism secondary to adrenal nodular hyperplasia. Outstanding quality-of-life, disease control and fertility were achieved.

## Background

Classic congenital adrenal hyperplasia (CAH) is an autosomal recessive disease of adrenal steroidogenesis due to mutations of the *CYP21A2* gene, which encodes the 21-hydroxylase (21-OH) enzyme. 21-OH deficiency results in impaired glucocorticoid and mineralocorticoid production and androgen excess. Clinically, the most severe form of CAH is classified as classic and the milder form is known as non-classic. Within the classic form there are sub-classifications that relate to the degree of aldosterone deficiency. These range from the salt-wasting (SW) to the simple virilizing (SV) forms, with good genotype-phenotype correlation [1]. Overproduction and accumulation of cortisol precursors, such as 17-hydroxyprogesterone (17-OHP) and androstenedione, occurs and these precursors are shunted into the androgen synthesis pathway resulting in androgen excess [1]. The increased androgen secretion in CAH also results in an increase in progesterone during the follicular phase of the menstrual cycle that can lead to irregular menses, amenorrhea and/or infertility [2, 3].

All patients with classic CAH require glucocorticoid and mineralocorticoid replacement therapy. Optimal therapeutic regimens are difficult to achieve. Children are treated with the short-acting glucocorticoid, hydrocortisone, but various glucocorticoid regimens are used for adults. At the National Institutes of Health (NIH) Clinical Center, approximately one-third of adults receive hydrocortisone, one-third receive prednisone and one-third receive dexamethasone [4]. Similarly, Arlt et al. reported a cohort of 203 adult patients in the United Kingdom and found that females with classic CAH received prednisolone (48 %), dexamethasone (17 %) or hydrocortisone (20 %) [5]. Glucocorticoid treatment is meant to replace cortisol as well as suppress the hypothalamic-pituitary-adrenal axis to prevent adrenal hyperplasia and adrenal overproduction of androgens. The use of glucocorticoids in the management of classic CAH has been lifesaving, but in many patients it can be difficult to adequately suppress excess androgens without causing hypercortisolism and cushingoid features. It is far more difficult to suppress adrenal androgen production than to prevent adrenal crises and patients often suffer from both glucocorticoid and androgen excess [6].

Although adrenalectomy as a treatment option for CAH has been proposed, it has only been performed in select cases [7]. At the NIH over 350 patients with CAH are being followed in a Natural History Study [4]. Two female patients have undergone adrenalectomy, one previously reported [7]. We report here our second case, a 21 year old female with classic SV CAH and refractory hyperandrogenism. She was treated with dexamethasone, fludrocortisone, an antiandrogen and oral contraceptive, and subsequently underwent bilateral laparoscopic adrenalectomy because of desired fertility.

## Case presentation

**Fig. 1:**
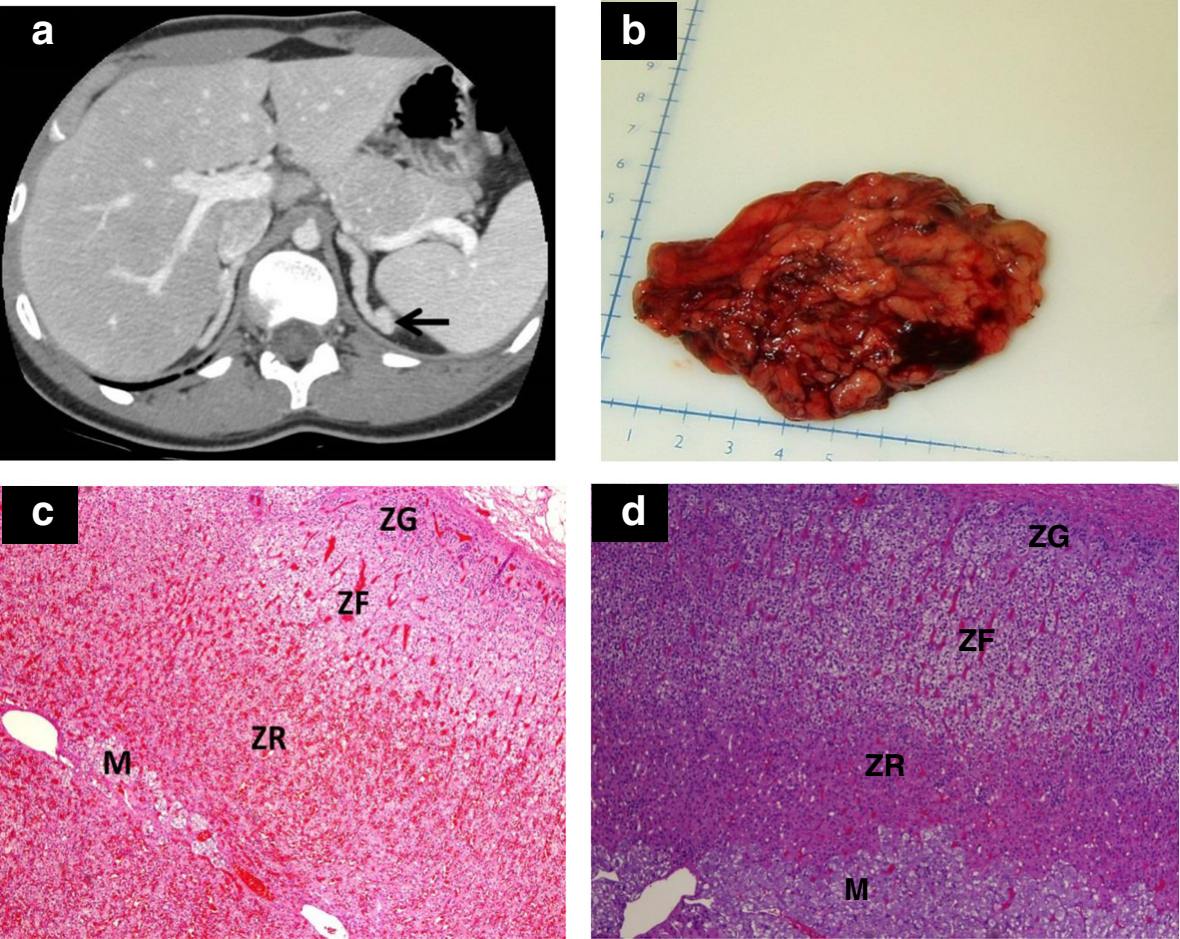
**a** Adrenal computerized tomography scan shows bilateral adrenal hyperplasia with a left adrenal gland nodule (arrow). **b** Hypertrophied right adrenal gland (weight 41.8 grams, normal adrenal weight 4 – 6 grams). **c** Histopathology of the classic simple virilizing CAH adrenal shows preservation of the adrenal cortex zonation (ZG, zona glomerulosa; ZF, zona fasciculata; ZR, zona reticularis) and the inner medulla (M) (**d**) Histology of a healthy unaffected adrenal gland shows the outer cortex with characteristic zones (Hematoxylin and eosin stain, magnification 4X)

Our patient was diagnosed with CAH at age two when she presented with clitoromegaly and increased growth velocity with a bone age of 5 years 6 months. Genetic testing confirmed the diagnosis of classic SV CAH (p.I172N, *CYP21A2* 30 kb deletion). She underwent clitoral reduction and vaginoplasty to correct a urogenital sinus. She first came to NIH at age 5, and showed a pattern of poor disease control as an adolescent. She had menarche at age 14 with subsequent secondary amenorrhea from age 15. Her weight fluctuated with BMI ranging from 22 to 29 kg/m^2^. She had consistently high laboratory values with a 17-OHP peaking at 52,900 ng/dL. Signs of virilization were controlled with the use of dexamethasone (250 mcg given twice daily), fludrocortisone (150 mcg daily), flutamide (250 mg given twice daily) and a combined oral contraceptive pill. These medications were well tolerated, liver function tests were monitored and remained normal; however, regular menses did not resume. At age 19, she married and desired fertility. Although virilization did not progress while receiving flutamide, androgen levels remained elevated (total testosterone range 160 – 283 ng/dL; normal 8 – 60 ng/dL) and could not be adequately suppressed without inducing significant cushingoid effects. Adrenal computerized tomography scan showed bilateral moderately hyperplastic adrenals with a left adrenal gland nodule (Fig. 1a). Bilateral adrenalectomy was considered as her androgens became increasingly difficult to suppress as well as her concern regarding fertility and a desire to start a family. A Bioethics consult was obtained and the pros and cons of bilateral adrenalectomy were discussed with the patient and her family independent of the primary team. The procedure was approved by the *Eunice Kennedy Shriver* National Institute of Child Health and Human Development Institutional Review Board. Written informed consent was obtained under the Natural History Study of Excess Androgen (NCT00250159, https://clinicaltrials.gov/ct2/show/NCT00250159).

A bilateral laparoscopic adrenalectomy was performed at age 21. The right adrenal gland weighed 41.8 grams (Fig. 1b) and the left gland 45.5 grams (normal adrenal gland weight 4 – 6 grams) (Fig. 1c). Her adrenal glands, despite generalized hypertrophy, maintained zonation with a small area of distinct medulla (Fig. 1d), consistent with her SV genotype. The patient was given 100 mg of intravenous hydrocortisone preoperatively and received stress doses of hydrocortisone for 2 days. Beginning on post-operative day 3, she was treated with hydrocortisone 20 mg in the morning and 10 mg in the evening and 100 mcg of fludrocortisone daily. On post-operative day 8, adrenal steroids (17-OHP, androstenedione and testosterone) were undetectable. She had a reduction of the evening dose of hydrocortisone to 5 mg in 6 weeks and was treated with an oral contraceptive for 10 months. After cessation of the oral contraceptive she became pregnant within 6 months. Since the surgery, she has had a total of 3 pregnancies, all within 6 months of trying to conceive. All three children were full-term, healthy and screened negative for CAH by hormonal testing. Follow-up 6 years later at age 27 revealed overall excellent health with a BMI of 25.1 kg/m^2^, no evidence of adrenal rest tissue based on hormonal testing, and above average quality-of-life on the 36-item short-form health survey (norm based scores across all eight domains on the 36 item short-form health survey ranged from 55.3 to 63.3, above the general population mean of 50). Since her adrenalectomy, she has not experienced an adrenal crisis.

## Discussion

Our case highlights the disease related morbidities and significant challenges of managing adult females with classic CAH. Our patient’s weight loss, lack of adrenal crisis and improved quality-of-life 7 years after surgery supports adrenalectomy as a therapeutic option in select cases, and especially in the treatment of female infertility. Suboptimal response to medical management of her CAH and a strong desire for fertility led to the consideration of surgery.

Our patient suffered from a combination of glucocorticoid excess and hyperandrogenism during adolescence. Her course was complicated by poor compliance, a common occurrence in adolescents with a chronic condition requiring daily medication [8, 9]. Management of adolescents with classic CAH is particularly challenging and focuses on control of androgen excess while avoiding cushingoid side effects of overtreatment. Prevention of long-term adverse outcomes, such as suboptimal sexual health, infertility and tumor formation, is of utmost importance [8]. Adrenal hyperplasia, nodularity and tumor formation occur with long-standing poorly controlled disease and if not prevented can complicate disease management, as seen with our patient presented here.

The desire to avoid high dose glucocorticoids in order to achieve adrenal androgen suppression led to our use of the anti-androgen flutamide and a combined oral contraceptive pill. The use of this therapeutic approach to control the signs and symptoms of hyperandrogenism was successful, but the long-term success of this therapy is unknown because our patient eventually desired fertility. To address the androgen excess feature of CAH, peripheral blockade of androgens and androgen synthesis inhibitors have been used in clinical trials in prepubertal children with classic CAH and in a phase 1 study of adult women with classic CAH respectively, but this approach is not considered standard therapy [10, 11]. However, the use of an oral contraceptive and the antiandrogen spironolactone is commonly used in the treatment of non-classic CAH, the mild form of the disease [12]. We did not use spironolactone in this patient with classic CAH due to the diuretic effect of spironolactone and because it is a weaker anti-androgen than flutamide. Flutamide acts to inhibit androgen activity and oral contraceptives are protective of the ovaries from becoming polycystic and androgen secreting. A study of flutamide administration in 8 patients with polycystic ovary syndrome showed an improvement in hirsutism, a decrease in androgen levels and a reduction in ovarian volume possibly due to flutamide’s effects on androgen biosynthesis in ovarian theca cells [13].

Adrenalectomy has been considered as a prophylactic treatment option for classic CAH [7, 14], but our case does not support this approach. A long term study of a 3 year old with SW CAH due to a double null mutation in *CYP21A2* who underwent prophylactic adrenalectomy revealed normal growth and development due to management with lower dose glucocorticoid replacement post adrenalectomy [15]. But caution should be used because genotype does not always predict adult phenotype [16]. Patients with salt-wasting CAH may do well as adults, while patients with simple virilizing CAH, such as the patient described here, may suffer from multiple co-morbidities. The distinction between SW and SV, although sometimes useful in the management of young children, may be unimportant in the management of adults with classic CAH [17]. Our patient is an example of this phenomenon. The factors that contribute to the development of adult co-morbidities require further study.

A common concern regarding adrenalectomy in patients with CAH is not the surgical procedure itself but the long-term risks following removal of the adrenals. Most important is the concern regarding the mortality risk and the potential protective function of the adrenals in CAH, which when stressed may produce small amounts of cortisol. There is also an observed decrease in blood pressure after adrenalectomy which may reflect a loss of mineralocorticoid protective effects against volume depletion [17]. In SW CAH, the adrenal glands are unable to produce sufficient amounts of cortisol and epinephrine and thus surgical removal of the adrenal glands most likely does not increase the risk of an adrenal crisis [18]. However, our patient had SV CAH and therefore would be expected to have some endogenous cortisol and epinephrine secretion. Indeed, her adrenal glands, despite generalized hypertrophy, maintained zonation with a small area of distinct medulla. Despite having a less severe genetic mutation and less developmental impairment of the adrenal gland, several years of noncompliance induced adrenal hypertrophy leading to a state of excess adrenal androgen production which was difficult to control with medical management alone.

The outcome after surgery with respect to quality-of-life, disease control and fertility indicates that bilateral adrenalectomy was a positive therapeutic option for our patient. Similarly, Ogilvie et al. reported two females with classic CAH who underwent adrenalectomy for infertility and became pregnant within 2 years [19].

Our patient continues on hydrocortisone and fludrocortisone replacement and is being followed annually. Given that our patient is doing well on glucocorticoid and mineralocorticoid replacement, we have not considered dehydroepiandrosterone (DHEA) supplementation, which would only be considered if she were to develop low libido, depressive symptoms or low energy despite optimized glucocorticoid and mineralocorticoid replacement [20]. In addition to monitoring glucocorticoid replacement based on clinical assessment, management includes measuring 17-OHP and androstenedione levels for evidence of adrenal rest tissue and measurement of plasma renin activity to assess mineralocorticoid replacement. A review of 18 cases of patient with CAH who had undergone adrenalectomy revealed that approximately one-third of patients had evidence of adrenal rest tissue activation following adrenalectomy leading to increased dosing of glucocorticoid replacement [7]. As suggested in the recent Endocrine Society Clinical Practice Guideline for management of primary adrenal insufficiency, measurement of ACTH to guide glucocorticoid replacement is not recommended [20].

Many patients who might be considered for adrenalectomy due to uncontrolled hyperandrogenism have a history of noncompliance, as seen in our patient. This habit of non-compliance must be carefully addressed prior to surgery and monitored after surgery because of the risk of experiencing a life-threatening adrenal crisis. Informed consent and patient education with a discussion of potential risks is essential. Close monitoring is warranted.

## Conclusions

The manifestations of classic CAH in adulthood are greatly influenced by disease control, prior therapy and compliance and acquired co-morbidities. Bilateral laproscopic adrenalectomy is not commonly performed as a therapeutic option for CAH. Concerns include surgical risk, loss of protective adrenal function and risk of recurrent virilization due to adrenal rest [12]. However, bilateral adrenalectomy was successful for our patient with classic SV CAH and may be considered as a treatment option for female patients with classic CAH with difficult to control hyperandrogenism, adrenal nodular hyperplasia and infertility.

## Consent

Written consent was obtained from the patient. A copy of the written consent is available for review from the Editor of the Journal.

## Abbreviations

17-OHP: 17-hydroxyprogesterone
21-OH: 21-hydroxylase
CAH: congenital adrenal hyperplasia
NIH: National Institutes of Health
SV: simple virilizing
SW: salt-wasting
